# Effects of Music Therapy in the Reduction of Pre-Meal Anxiety in Patients Suffering from Anorexia Nervosa

**DOI:** 10.3390/brainsci12060801

**Published:** 2022-06-19

**Authors:** Enrico Ceccato, Cristina Roveran

**Affiliations:** 1 Department of Mental Health, San Bortolo Hospital, 36100 Vicenza, Italy; 2 The Giovanni Ferrari Music Therapy School, 35100 Padua, Italy; cristina.roveran@sonepar.it

**Keywords:** MT, AN, eating disorder, music, adolescents

## Abstract

Literature reviews appear to indicate that Music Therapy (MT) may instil a sense of empowerment and generate feelings of renewed self-confidence, distracting subjects who follow this type of intervention from negative thoughts and, generally, helping patients suffering from Anorexia Nervosa (AN) to redevelop or rediscover their identity. The purpose of the study reported in this paper is to investigate whether MT proposed before an evening meal is capable of decreasing pre-meal anxiety in adolescents suffering from AN who follow the Day-hospital Treatment Programme at the San Bortolo Hospital of Vicenza (Italy). A total of 24 patients participated voluntarily in once-weekly sessions of group-based MT conducted by a qualified music therapist over a period of six months. Before evening meals on Monday, Tuesday and Wednesday, pre-meal anxiety was measured using a self-report scale, and the MT group session occurred every Wednesday before the evening meal was consumed. MT activities were both active and receptive. It has been found that with respect to Mondays and Tuesdays, pre-meal anxiety was significantly lower on Wednesdays following participation in the MT group. MT is evidently capable of reducing pre-meal anxiety and may be adopted as a supportive element in treatment plans relating to patients with AN in a day-hospital treatment programme.

## 1. Introduction

Eating Disorders (EDs) represent a world-wide cause of psychiatric and physical morbidity and mortality. The overall incidence of EDs has been reported to have significantly increased over the last decade [1,2,3,4]. Moreover, the illness is affecting people at an increasingly younger age [3,4]. The prevalence of Anorexia Nervosa (AN) is estimated to be approximately 1% among women, with women being affected about 10 times more often than men [5]. 

According to the Diagnostic and Statistical Manual of Mental Disorders (DSM-5), subjects with AN present restrictive eating behaviour, a body weight that is significantly too low in terms of their age, sex and developmental trajectory, a fear of gaining weight and an anomalous perception of their body (body image disturbance) [6].

Anxiety and depression are often associated with eating disorders, particularly in adolescent and anorexic patients [7,8]. 

Moreover, research studies in literature appear to suggest there is a direct association between anxiety and restrictive eating behaviour and, in the case of patients with AN, a higher degree of pre-meal anxiety is associated with lower caloric intake [9,10]. 

Despite there being a significant association between anxiety and food intake, not many studies have been undertaken with a view to test the effects of reducing pre-meal anxiety.

Steinglass et al. [11] attempted to verify whether, by decreasing pre-meal anxiety through the use of an anxiolytic agent (Alprazolam), there would be greater caloric intake in patients with AN. The researchers observed that caloric intake did not differ with the administration of Alprazolam versus a placebo and, moreover, Alprazolam did not reduce anxiety but was associated with increased fatigue. 

In a recent study, Smith et al. [12] examined changes occurring in meal-related anxiety as a potential predictor of outcomes within the context of a family-based partial hospitalisation programme and suggested that reductions in meal-related anxiety may be an important predictor of outcomes within the sphere of family-based interventions. Various researchers have underlined that future investigations are required to examine whether the direct targeting of meal anxiety may enhance the outcome of therapy. 

With pre-meal anxiety established as a therapeutic target [9], mealtime support is typically supervised by inpatient staff [13]. It has been ascertained that mealtime assistance interventions included in a hospital rehabilitation programme based on Enhanced Cognitive Behaviour Therapy are able to determine weight normalisation in 86% of patients who have completed treatment [14].

Apart from the relative psychological aspects, anxiety involves complex mechanisms and various neuronal circuits [15]. Several areas involved in the modulation of anxiety have been identified with respect to the processing of anxiogenic stimuli. These include the thalamus, which acts as a primary link between the exteroceptive receptor systems and cortical areas, and the amygdala, which is responsible for the acquisition and expression of conditioned fear through a short, automatic and involuntary path and a long path, which involves the processing of stimuli by the cortex [15]. The efferent pathways of the anxiety-fear circuit will trigger an autonomic response involving the autonomic nervous system that generates somatic symptoms, such as higher blood pressure and an increased heart rate, sweating, pupillary dilation and urinary and gastrointestinal problems [16]. 

Both music-making and music-listening therapy will influence the activity of the autonomic nervous system [17,18], and some studies suggest that repeated music-making and listening may have short-term effects linked to acute reductions in stress and anxiety [19]. Moreover, a long-term effect on the autonomic tone has been identified [20,21,22]. 

Active and receptive forms of Music Therapy (MT), with both group and individual sessions, have been administered to patients with EDs. Qualitative studies and case studies would appear to underline the notion that MT may engender a sense of empowerment, feelings of renewed self-confidence, distraction from negative thoughts and a sense of autonomy in patients suffering from ED [23,24,25,26,27]. 

Bibb et al. have demonstrated that a MT session occurring after lunchtime significantly lowers post-meal anxiety in inpatients and outpatients who present AN [25,28]. 

In both studies, the MT sessions referred to were conducted by a music therapist who encouraged patients to actively engage in the group activities by singing, listening to songs, sharing thoughts on music and writing songs themselves [25,28]. Whilst the efficacy of MT interventions with respect to the reduction of post-meal anxiety in the inpatient group was compared to conventional therapeutic support [25], there was no control group in the outpatient study [28]. In the study conducted by Cardi et al. on the impact of classical piano music on food consumption [29], it is noted that inpatients who listened to piano music at mealtimes reported lower levels of distress and consumed a higher quantity of food compared to control subjects (vodcast group). The aim of this study was to investigate whether MT is capable of reducing pre-meal anxiety in patients with AN.

## 2. Materials and Methods

### 2.1. Participants

The sample included 24 patients with a diagnosis of acute AN, all of whom were females, attending the daily therapy programme at the Eating Disorder Unit of the San Bortolo Hospital in Vicenza. The Eating Disorder Unit includes patients aged 12 and over who present severe AN and have not been able to recover through outpatient treatment. The Eating Disorder Unit can accommodate 12 people, and each individual programme, which may be renewed, will last for periods of 3 to 6 months. A collaborative multidisciplinary and patient-centred approach is adopted, which focuses on individualised treatment [30]. 

Patients who attend the Eating Disorder Unit are involved in multiple occupational and rehabilitation activities, of both the individual and group-based type, and mealtime support is provided by a team member during each midday meal [31,32]. In the case of anorexic patients, weight recovery is important for the resolution of complications associated with malnutrition and for clinical stabilisation [33,34]. The aim of the rehabilitation process is to normalise the patient’s eating behaviour, restrict all symptomatic manifestations and allow each subject to acquire a ‘normal’ eating experience [33,34]. Within our unit, meals are served in a dining room, and patients eat together at the dining tables. On each occasion, staff members (usually a dietician or a nurse) organise and manage mealtime activities to facilitate meal completion and to achieve normalised forms of eating behaviour. The meal support process consists in assisting and monitoring patients as they consume food, with the aim of removing obstacles which impede them from resuming an adequate form of behaviour [34]. The use of cellular devices is not permitted during lunch and the patients are not allowed to go to the toilets. They are required to try to consume all of the contents brought to them on a tray, and, as a general rule, they are dissuaded from talking about food. At our therapy, centre staff members do not sit down and eat together with the patients but try to encourage dialogue and socialisation during the course of each meal. They support the patients and exercise implicit control over the topics of conversation, actively participating and encouraging communication between everyone present in the room. At the same time, staff members have to monitor the amount of food consumed by each individual patient, as food rituals may occur (eating very slowly, chopping the food into small pieces and scattering it over the plate, the exclusion of condiments, etc.). The staff members deploy specific skills aimed at promoting a reduction of anxiety with respect to nutrition and increasing a feeling of control related to the body-weight normalisation process. The real purpose of meal-assistance activities is not to control what a patient consumes in terms of calories, but to offer emotional support during the most difficult moments of the patients’ rehabilitation. Staff members also educate patients to eat without suffering the effects of ‘food worries’ and, if such concerns arise during a meal, their attention is drawn towards the goals agreed upon, helping them to return to a healthy form of eating behaviour [35].

All patients participating in the study met the full diagnostic criteria for AN according to the DSM-5 [6]. 

Criteria for exclusion:

(1) male gender;
(2) age under 14;
(3) current or lifetime neurological diseases;
(4) mental impairment or learning disabilities;
(5) bipolar disorder or schizophrenia spectrum disorder;
(6) history of drug/alcohol dependence.

Table 1 is a summary of demographic and clinical characteristics of patients who attended the MT sessions.

### 2.2. Procedure

From January to June 2021, for a total of 19 sessions, patients admitted to the Eating Disorder Unit participated in MT sessions lasting one hour (from 5.30 p.m. to 6.30 p.m.). The sessions were held every Wednesday before dinner was served (at 6.45 p.m.). 

The Eating Disorder Unit provides evening meals only from Monday to Wednesday. On Monday, the patients participate in creative activities (decoupage workshops); on Tuesdays, the patients participate in group psychotherapy sessions with a cognitive-behavioural orientation. On Thursdays and Fridays, the Unit closes at 4 p.m. The MT sessions have always been attended by numerous patients, with an average of 10 persons present. The MT activities avoided any strict impositions regarding attendance, and any girls who felt better were discharged and replaced by others presenting worse clinical conditions. 

The sessions were all conducted in a multipurpose room where various rehabilitation activities were carried out. The room was not soundproofed but was sufficiently quiet and well-lit and had ample spaces that allowed the participants to easily move around.

The entire range of activities at the centre is designed for all patients attending treatment sessions; therefore, for this small-scale preliminary study, we did not set up a control group for the research, preferring to measure the presence of anxiety preceding an evening meal also on days when the MT activities did not take place. 

Pre-meal anxiety was consistently measured from January to June on Monday, Tuesday and Wednesday evenings using the Anxiety Thermometer (AT) [36,37,38]. The scale was delivered by a nurse of the Eating Disorder Unit just after the end of the MT group and before evening meals. 

Patients provide an indication of their feelings using the AT every night as a routine procedure and were not informed of the objectives of the study. The nurses were also not informed of the purpose of the study, so the patients themselves, the nurses and the music therapist were entirely unaware of its aims. The only person fully cognizant of the goal of the research was the investigator himself, the psychologist of the Eating Disorder Unit. The head of the centre was also informed of the ongoing research. 

No specific written informed consent was required from the patients involved in the study, and an opinion on the part of the local ethics committee was not sought, as the tools used for the assessments are the same as those adopted in normal clinical practices and MT forms part of the Eating Disorder Unit activities, with respect to which patients provide their initial consent in writing. 

MT is one of the activities carried out at the Eating Disorder Unit, but the technique is not administered on a continuous basis. It is normally conducted by an external professional from September to June and suspended during the summer. Due to complications arising due to the COVID 19 pandemic, all of the activities entrusted to external professionals were suspended during 2020. The MT activities restarted in January 2021.

### 2.3. Measures

The AT was used to measure anxiety as self-reported by participants. The AT is a visual analogue scale in which participants rate their level of anxiety from 0 (none) to 10 (extreme). 

**Figure 1:**
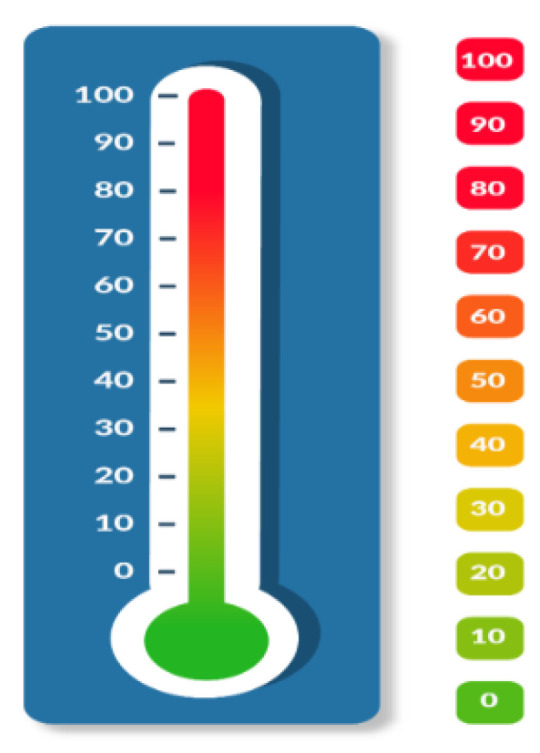
The anxiety thermometer (AT); we created this image following Mitchell’s et al. Indications [36].

The request was formulated as follows:

“Try to measure your degree of anxiety on a scale from 0 to 100, where 0 corresponds to ‘completely relaxed’ and 100 corresponds to ‘as much anxiety as possible’, Mark the number that best identifies your status on the thermometer (Figure 1).”

### 2.4. Treatment 

Following the indications of Robb, Carpenter and Burns, [39] we shall provide a description of the main features of the intervention. The group was guided by a trained Music Therapist without the presence of other health-care personnel. On the basis of our experience in MT, as applied to anorexic patients admitted to our centre [26], during each therapy session those participating were encouraged to engage in active and receptive MT activities and were asked to listen to music and also play, sing and write songs. 

The sessions were divided into three phases:

(1) warm-up phase: short preliminary activities to distract participants from dysfunctional thoughts and focus attention on the activity to be performed;
(2) central phase: focus on the topic of the session, implemented through MT activities;
(3) conclusion: verbal discussion on what happened during the session.

In the central phase, improvisational activities were carried out (a set of Orff instruments was present in the room). The improvisation started with simple rhythmic structures in 4/4 timing without the use of the voice. Although prompted by the music therapist, this was a difficult endeavour. In two cases, when the voice was included in the improvisation, the volume of the vocal element was very low. The patients were provided with pencils and markers and were invited to express their thoughts on sheets of paper and posters. The therapist proposed music for the receptive phases, this being an intervention planned before the session was held. The choice of music was based on knowledge acquired by the therapist with regard to the possibility of obtaining certain results, such as relaxation. A portable PC was used and positioned at the side of the room. The therapist adjusted the volume of the music according to its purpose at a particular time. If the aim was to facilitate relaxation, the volume would be kept low; if the intention was to stimulate emotions, the volume would be turned up.

In some cases, the patients were asked to choose and propose songs themselves which might represent their mood. In this case, the songs would differ from those containing significant textual elements. Participation in the choice of songs might allow for the representation of occurrences of relational closeness during their adolescence [40]. During the sessions, a song entitled ‘Do it for you’ was created.

During some sessions, musical psychodrama techniques [41] were adopted. In this case, music was used to involve the participants and led to the selection of a protagonist of the psycho-dramatic work itself. 

After each session, a personal observation form was completed, and a ‘logbook’ was compiled by the music therapist, which were then shared with the psychologist in order to discuss and monitor the intervention.

A humanistic approach was adopted [42]. 

### 2.5. Statistical Analyses

For the statistical analysis, a paired-samples *t*-test was used in order compare the mean values.

## 3. Results

There was a significant difference both between the level of anxiety measured on Monday and the level measured after the MT group session held on Wednesday, with a score of t_(gl)_ = 3.847_(23)_, and between the level of anxiety measured on Tuesday and the level of anxiety measured after the MT group session on Wednesday, with a score of t_(gl)_ = 3.053_(23)_.

In particular, this analysis reveals a significantly lower level of pre-meal anxiety on Wednesday after the MT group session compared to the other two days (Table 2).

As previously mentioned, the pre-meal anxiety levels were measured on Monday, Tuesday and Wednesday before each evening meal, and on Wednesday evenings, after the MT group, the level of anxiety was always lower than on the other evenings. 

## 4. Discussion

The aim of this study was to investigate whether MT can reduce pre-meal anxiety in patients with AN. The results obtained were positive and support the use of MT in a daily programme for the treatment of AN. Intervention was found to be feasible and confirms that MT is well received by patients with AN [43,44].

MT adopted with subjects who present AN involves considerable difficulties. The condition which characterises this disorder includes a mood that is often deflected, poor motivation with respect to participation in any activity and, thus, a low level of willingness to become involved. At some of the Wednesday sessions, the patients would arrive already exhausted by the activities they had been engaged in during the day, and this made it difficult to invite them to participate in certain exercises. Other difficulties arose in relation to a degree of variability identified in the individual state of members of the group. As stated above, girls who felt better and were discharged were replaced by girls presenting worse clinical conditions, and this repeatedly posed a challenge in terms of the necessity to generate a positive atmosphere by integrating the new participants. 

As this was an ‘open’ group, in which, over time, the participants would be replaced, the focus group method could not be adopted at the end of the activity, nor was it possible to compile a qualitative overview of the patients’ impressions regarding the MT activities. However, the music therapist did constantly update his own personal diary after each session, recording his observations and adding notes referring to comments made by the patients during group sessions. From these notes, it has emerged that during the full period of intervention, from the very first to the final session, a great change occurred, especially with respect to the involvement of patients. Initially, they were very closed and struggled to express their thoughts or emotions, and sometimes it was necessary to repeat a request several times, as they were unable to comprehend what was required of them, whereas at the end of the series of sessions, a brief invitation would be sufficient to get them started. It was also ascertained that MT introduced a good form of support in their treatment because it could help them to express their emotions and experience their feelings in a more personal way. Comments such as “For the first time I feel that I have some friends… real friends…”; “I finally felt that someone really understood me…”; “I didn’t think that by listening to a song you would be able to understand so much about me…”, highlight the manner in which patients succeeded not only in expressing their own fragility but also managed to create deeper bonds with those who, in that moment, accompanied them along the path they were now following. Reports even indicated that some girls would subsequently make spontaneous use of music they had heard or texts they had read during the sessions in order to relax or relive emotions they had experienced during the group therapy. These notes reinforce our idea that MT is not only a pleasant activity but also a method whereby each participant was able to become more aware of their inner self, allowing them to liberate their body and express their emotions. 

The principal result of the quantitative analysis was that MT reduced pre-meal anxiety, offering an indication of ways in which MT may be used in the treatment of AN. We imagine that having less pre-meal anxiety means being able to follow dietary guidelines more calmly and potentially reduces the need for specialist assistance during meals. With respect to patients with AN, the capacity to intervene in a manner that will reduce anxiety before evening meals may constitute a very important contribution to the recovery process, which will necessarily involve weight gain. It would certainly be worth undertaking further studies to investigate such potential results.

Comparing the pre-meal activities of the sessions held on Monday and Tuesday with those practised during the MT sessions, the fact that the latter decreased pre-meal anxiety made us reflect. In any case, a comparison of the effects of these activities on pre-meal anxiety was not the intention of this study. We felt that the pre-meal anxiety measured on Mondays and Tuesdays would represent a standard measure to which the degree of anxiety experienced on Wednesday could be compared beyond any pre-meal activity. The activities occurring on Monday and Tuesday were not specifically determined to reduce pre-meal anxiety, whereas the Wednesday session had this precise purpose. Furthermore, the possibility that MT may in fact have had an interactive effect with the other activities cannot be excluded.

The result of this study is in line with the findings of two recent reviews in which it is sustained that listening to music appears to be effective in the reduction of anxiety in a large range of clinical populations [45,46]. In particular, Harney et al. point out that the significant reduction in anxiety may be linked to the emotional regulatory effects of this activity. Emotional regulation is commonly cited in relative literature as the main function of listening to music [47,48]. Furthermore, a particular study in which both active and receptive MT is adopted indicates that MT was effective in reducing anxiety and depression levels in GAD patients [49]. The authors suggest that a possible explanation of the positive effect of several active MT sessions is that when we engage in intense movements of the body in a creative manner, we stimulate the release of physical and psychological tension. 

We also believe that MT may have had a greater influence in reducing anxiety because it results in a more intense stimulation of emotional responses on the part of patients, and this may be a focus of further research.

Despite these interesting findings, the study did present some limitations. Due to the fact that the size of the sample was quite small, we were unable to differentiate between the subtypes of AN (restricting vs. binge-eating/purging), the duration of the illness and the stage of recovery. Moreover, the patients were all female. Thus, the results cannot be generalised to males. From our own perspective, a lack of generalisability is a fundamental concern with respect to research related to MT practised with a view to assisting people with eating disorders and, more specifically, with regard to AN. Randomized Controlled Trials would be required. 

## 5. Conclusions

In summary, the data suggest that MT is a feasible method and may be very useful to reduce pre-meal anxiety in patients with AN who attend a daily programme in an Eating Disorder Unit. 

However, considering the small number of subjects and the lack of control groups, the experimental design could be further enhanced in the future to validate the findings. 

Furthermore, future research should be conducted to explore how the process of music therapy actually works.

## Figures and Tables

**Table 1:** Demographic and clinical characteristics of the sample.

	Mean	SD	Min	Max
Age (years, months)	17.43	3.29	14	25.6
Education (years)	12	3	8	18
BMI	15.72	2	13.3	20.3
Illness duration (months)	16.87	12.96	2	60
MT sessions attended	8.92	5.82	1	17

**Table 2:** Results of *t*-test, significance: *p* < 0.05.

	**Mean**	**N**	**SD**	**Average SD**	***t***	**gl**	**Sign.** **(Two-Tailed)**
Pre-meal anxiety Tuesday	55.6409	24	30.83210	6.29358	3.053	23	**0.006**
Pre-meal anxiety Wednesday	52.0625	24	31.42944	6.41551			
Pre-meal anxiety Monday	57.7339	24	30.27079	6.17900	3.847	23	**0.001**
Pre-meal anxiety Wednesday	52.0625	24	31.42944	6.41551			

## Data Availability

The principal data generated or analysed during this study have been included in this article. Further enquiries may be submitted to the corresponding author.
